# Appendectomy and colorectal cancer: a mini review from the perspective of gut microbiota and mucosal immunity

**DOI:** 10.3389/fcimb.2026.1859485

**Published:** 2026-06-10

**Authors:** Shuai Wang, Jingbo Lv, Li Wang, Xueyuan Cao

**Affiliations:** 1 Department of Gastric and Colorectal Surgery, General Surgery Center, The First Hospital of Jilin University, Changchun, Jilin, China; 2 Department of Cardiovascular Medicine, The First Hospital of Jilin University, Changchun, Jilin, China

**Keywords:** appendectomy, colorectal cancer, Fusobacterium nucleatum, gut microbiota, mucosal immunity, tumor associated macrophages

## Abstract

The vermiform appendix was long considered a vestigial organ, yet accumulating evidence now supports its role as a component of gut associated lymphoid tissue and as a niche involved in microbial homeostasis and mucosal immune regulation. Against this background, whether appendectomy influences colorectal cancer (CRC) development and progression has become an important question at the intersection of oncology, microbiology, and immunology. Current epidemiological evidence does not support a simple model in which appendectomy uniformly increases long term overall CRC risk. Earlier observational studies, particularly from some Asian and American databases, suggested an increased risk in the short to intermediate period after surgery, but more recent prospective cohorts, molecular pathological epidemiology studies, and Mendelian randomization analyses indicate that this signal is highly dependent on time window and study design, and is more likely to reflect reverse causation and detection bias in the early postoperative period. At the same time, mechanistic and translational studies provide a biologically plausible framework, suggesting that appendectomy may be associated with changes in gut microbial communities, epithelial barrier function, inflammatory signaling, and tumor-associated macrophage-related immune programs, although much of the current evidence remains associative in humans or experimental in nature. Notably, appendectomy may not affect all CRCs in the same way, but may instead influence selected tumor locations and microbe related molecular subtypes, particularly Fusobacterium nucleatum (F. nucleatum) associated tumors. Overall, the relationship between appendectomy and CRC should be interpreted as a complex microbiota and immunity related issue rather than a simple unidirectional carcinogenic model. Future studies should clarify the true effects across different time windows, tumor subtypes, and immune ecological settings.

## 1 Introduction

The possible link between appendectomy and colorectal neoplasia has been discussed for decades. In 1964, McVay Jr. first investigated the connection between the appendix and neoplastic disease, establishing one of the earliest landmark works in this field (McVay, 1964). Subsequent observations by Berndt and Howson kept the hypothesis alive by asking whether appendectomy might be followed by altered cancer risk (Berndt, 1970; Howson, 1983). However, those early studies were limited by retrospective design, modest sample size, and uncertainty regarding temporality. In particular, it was often impossible to determine whether appendicitis preceded colorectal tumor development, whether an occult colonic lesion triggered appendiceal inflammation, or whether surgery itself had any lasting biological effect (Mellemkjaer et al., 1998; Cope et al., 2003; Lai et al., 2006).

Interest in the topic reemerged when the biological view of the appendix changed. The appendix is no longer regarded as a functionless remnant. It contains abundant lymphoid follicles, has features of gut associated lymphoid tissue, and may contribute to local antigen sampling, immune tolerance, secretory IgA related responses, and microbial recovery after ecological disruption (Randal Bollinger et al., 2007; Guinane et al., 2013; Kooij et al., 2016; Seikrit and Pabst, 2021). In parallel, the gut microbiota has become central to modern models of colorectal carcinogenesis, with mounting evidence that dysbiosis, barrier impairment, microbial toxins, inflammatory myeloid signaling, and altered T cell responses can shape tumor initiation and progression (Sears and Garrett, 2014; Garrett, 2015; Brennan and Garrett, 2016; Sun and Kato, 2016; Raskov et al., 2017; Tilg et al., 2018; Montalban-Arques and Scharl, 2019; Wong and Yu, 2019; Cheng et al., 2020; Janney et al., 2020). These developments have shifted the central question. The issue is no longer merely whether appendectomy increases overall CRC risk, but whether removal of an immune active and microbiota associated organ changes the ecological and immunological context in which colorectal tumors arise or evolve.

As a key immune ecological interface between the host and gut microbiota, the appendix exerts functional roles that extend far beyond crude analyses of overall cancer incidence. This mini review consolidates current evidence around three interconnected themes: the actual findings from epidemiological data, the contributions of the appendix to mucosal immunity and microbial homeostasis, and the immune and microbiota−dependent mechanisms underlying the time−dependent and subtype−specific associations reported in recent studies.

## 2 The appendix as an immune and microbial niche

Understanding the appendix as an immune active organ is essential for interpreting the current evidence. Kooij and colleagues summarized data showing that the vermiform appendix contains abundant lymphoid tissue and participates in local immune regulation rather than serving as a useless remnant (Kooij et al., 2016). Related work in inflammatory bowel disease has also suggested that the appendix may influence intestinal immune programming beyond what was traditionally assumed (Sahami et al., 2016). At the mucosal level, these functions can be linked to the geography of IgA induction and intestinal lymphoid specialization. Secretory IgA is crucial for pathogen containment, commensal shaping, and maintenance of epithelial tolerance, and its induction depends on anatomically organized mucosal immune structures (Macpherson et al., 2008; Seikrit and Pabst, 2021). The appendix is not the only such structure, but its location at the cecal pole, its dense lymphoid content, and its favorable architecture for microbial biofilm formation make it biologically plausible that it could serve as a local immune ecological hub.

Bollinger and colleagues proposed the influential “safe house” concept, in which the appendix acts as a protected reservoir for commensal biofilms that can help repopulate the colon after diarrheal or infectious disruption (Randal Bollinger et al., 2007). Human microbial studies further support the notion that the appendix harbors a distinct microbial composition rather than being a passive extension of fecal content (Guinane et al., 2013). Thus, appendectomy may remove not only an inflamed organ, but also a site with meaningful immunological and ecological functions. This concept provides a coherent framework for understanding why appendectomy could influence CRC biology even if it does not produce a uniform increase in long term overall incidence.

## 3 Epidemiological evidence linking appendectomy and CRC

Early studies did not establish a consistent long term cancer signal. A Danish registry study published in 1998 did not support a stable association between appendectomy for acute appendicitis and overall malignancy risk (Mellemkjaer et al., 1998). A Swedish childhood and adolescent cohort also failed to demonstrate a broad long term cancer hazard that could convincingly be attributed to appendectomy itself (Cope et al., 2003). Still, clinical observations raised concern that acute appendicitis in older adults might sometimes be the first manifestation of an occult colonic lesion. Lai and colleagues reported that colon cancer could initially present as appendicitis, particularly in the cecum or right colon (Lai et al., 2006).

This interpretation gained support from later population based work. In a Taiwanese study, Wu and colleagues reported that appendectomy was associated with a higher subsequent incidence of CRC, with the highest risk during the period 1.5 to 3.5 years after surgery (Wu et al., 2015a). The same group also showed that appendicitis itself could function as an early manifestation of later malignancy (Wu et al., 2015b). A large Swedish registry based study found altered gastrointestinal cancer risk patterns after appendectomy, although the associations were not uniform across sites or age groups (Song et al., 2016).

Several studies now strongly support the idea that acute appendicitis in middle aged and older adults can act as a warning sign for underlying colonic neoplasia. A systematic review and meta analysis focusing on patients older than 40 years found an increased incidence of right sided colon cancer after acute appendicitis (Hajibandeh et al., 2020). Similar conclusions were reached by a retrospective study from New Zealand and by a nationwide French population based study, both of which showed that the excess colon cancer signal was strongest in the early period following appendicitis and was predominantly located in the cecum and right colon (Shine et al., 2017; Viennet et al., 2023). A more recent systematic review on post appendectomy colorectal screening reported meaningful rates of adenoma and occult carcinoma detection in adults older than 40 years, again with a predominance of right sided lesions (Esposito et al., 2024). These findings are clinically important because they suggest that, in a substantial subset of patients, acute appendicitis or appendectomy is better interpreted as a diagnostic clue than as a carcinogenic exposure. Importantly, the current evidence does not support routine colonoscopic evaluation for all adults after appendicitis or appendectomy. Rather, a more appropriate interpretation is that post-event colonoscopic assessment may be considered selectively in adults older than 40 years, particularly in those aged 45 years or above who are due or overdue for age-appropriate CRC screening, and in patients with alarm features such as persistent bowel symptoms, iron deficiency anemia, weight loss, suspicious imaging findings, or an atypical clinical course. This approach is more consistent with a targeted diagnostic strategy than with universal post-appendectomy screening.

When attention shifts from short term diagnostic association to long term overall risk, the evidence becomes notably more conservative. A pooled analysis of three large prospective cohorts did not identify a meaningful increase in long term CRC incidence after appendectomy (Rothwell et al., 2022). A 2024 study that extended the same framework to include adenoma outcomes likewise found no evidence for an adverse long term association with either CRC or adenoma (Zhang et al., 2024). Although the 2023 meta analysis reported an overall pooled increase in CRC risk after appendectomy, the investigators also showed marked heterogeneity across regions, with no stable excess risk in European studies (Liu et al., 2023). The two Mendelian randomization analyses published in 2024 further weakened the argument for a direct causal effect by finding no convincing genetic evidence that appendectomy causes CRC (Wang et al., 2024; Wei et al., 2024).

The most conceptually important recent advance came from molecular pathological epidemiology. Kawamura and colleagues showed that appendectomy was not associated with increased long term overall CRC incidence, but was associated with a lower incidence of F. nucleatum positive CRC (Kawamura et al., 2025). This observation shifts the way the field should think about the problem. The key question may not be whether appendectomy increases CRC in general, but whether it changes the probability of developing specific microbiota linked molecular subtypes. That interpretation is more consistent with the heterogeneous epidemiological record and with the emerging biological role of the appendix in immune and microbial homeostasis (**Table 1**).

**Table 1:** Representative epidemiological and mechanistic studies on appendectomy and CRC.

Study	Design/population	Main exposure/focus	Key finding	Main implication
McVay Jr., 1964 (McVay, 1964)	Narrative clinical/pathophysiological discussion	Appendix and neoplastic disease	Early article that framed the appendix as potentially relevant to neoplastic processes rather than an entirely negligible organ	Historical starting point of the field; hypothesis-generating rather than causal evidence
Mellemkjaer et al., 1998 (Mellemkjaer et al., 1998)	Population-based registry study, Denmark	Appendectomy for acute appendicitis	Did not support a stable long-term overall cancer risk increase after appendectomy	Early large-scale evidence arguing against a simple universal carcinogenic effect of appendectomy
Cope et al., 2003 (Cope et al., 2003)	Nationwide cohort, Sweden	Childhood/adolescent appendectomy	No clear long-term broad cancer hazard attributable to appendectomy	Weakens the argument for a straightforward lifelong cancer-promoting effect
Lai et al., 2006 (Lai et al., 2006)	Retrospective clinical study, 1,873 patients	Appendicitis as first manifestation of colon cancer	Suggested that appendicitis can be the first presentation of occult colon cancer	Supports reverse causation, especially for right-sided lesions in adults
Wu et al., 2015 (Wu et al., 2015a)	Population-based cohort, Taiwan	Appendectomy and subsequent CRC	Reported increased CRC incidence after appendectomy, with risk concentrated in the first few postoperative years	One of the strongest studies supporting a short/intermediate-term epidemiological signal
Wu et al., 2015 (Wu et al., 2015b)	Population-based cohort, Taiwan	Appendicitis and subsequent malignancy	Appendicitis may be an early manifestation of subsequent malignancy	Reinforces that some observed associations may reflect occult cancer rather than causal surgery effects
Song et al., 2016 (Song et al., 2016)	Register-based nationwide cohort, Sweden	Appendectomy and gastrointestinal cancers	Associations varied by site and subgroup; no uniform pattern across gastrointestinal cancers	Suggests heterogeneity by tumor site and population context
Hajibandeh et al., 2020 (Hajibandeh et al., 2020)	Systematic review and meta-analysis	Acute appendicitis in adults older than 40 years	Increased incidence of right-sided colon cancer after acute appendicitis	Supports postoperative colonic evaluation in selected older adults
Rothwell et al., 2022 (Rothwell et al., 2022)	Pooled analysis of 3 prospective cohorts, Europe	Appendectomy and long-term CRC risk	No meaningful increase in long-term overall CRC risk	High-quality evidence against a stable long-term overall risk increase
Liu et al., 2023 (Liu et al., 2023)	Systematic review and meta-analysis, 22 studies	Appendectomy and CRC risk	Overall pooled OR 1.31; association not significant in Europeans, but positive in Asian and American studies	Suggests that the signal exists but is highly heterogeneous by region and study design
Viennet et al., 2023 (Viennet et al., 2023)	Nationwide population-based study, France	Acute appendicitis and subsequent colon cancer	Markedly increased colon cancer diagnosis shortly after acute appendicitis, especially within the first year	Strong support for reverse causation/diagnostic signal, especially for right colon cancer
Shi et al., 2023 (Shi et al., 2023)	Population cohort + metagenomics + experimental study	Microbiome changes after appendectomy	Appendectomy was associated with microbial dysbiosis, barrier dysfunction, and increased experimental tumorigenesis; the population cohort reported higher long-term CRC risk	Provides important mechanistic and translational evidence linking appendectomy to CRC-related biological changes
Esposito et al., 2024 (Esposito et al., 2024)	Systematic review	Colorectal screening after appendectomy in adults	Post-appendectomy screening in adults older than 40 years detected adenomas and occult cancers, many on the right side	Clinically supports targeted postoperative colon evaluation in selected adults
Zhang et al., 2024 (Zhang et al., 2024)	3 large prospective cohorts + meta-analysis	Appendectomy and CRC/adenoma risk	No adverse long-term association with CRC or adenoma; short-term signal more consistent with bias or reverse causation	Further weakens the long-term causal hypothesis
Wei et al., 2024 (Wei et al., 2024)	Mendelian randomization	Genetic liability related to appendectomy/appendicitis and CRC	Did not support a causal effect on CRC risk	Genetic epidemiology argues against simple causality
Wang et al., 2024 (Wang et al., 2024)	Mendelian randomization + meta-analysis	Appendectomy and gastrointestinal cancers	No genetic evidence for causality in European populations	Consistent with the view that observational signals may be confounded
Cai et al., 2021 (Cai et al., 2021)	Human gut microbiome study	Bacterial and fungal communities after appendectomy	Appendectomy was associated with altered gut bacterial and fungal communities, with durable effects particularly on fungi	Supports the appendix as a microbiota-associated niche
Kawamura et al., 2024/2025 (Kawamura et al., 2025)	Molecular pathological epidemiology	Appendectomy and CRC by tumor F. nucleatum status	Appendectomy was associated with lower long-term incidence of F. nucleatum-positive CRC, but not F. nucleatum-negative CRC	Key evidence that appendectomy may influence specific microbiota-linked molecular subtypes rather than all CRC equally
Liu et al., 2024 (Liu et al., 2024)	Clinical and experimental translational study	Appendectomy, prognosis, and TAMs in CRC	Prior appendectomy was associated with poorer CRC prognosis and depletion of M1-like macrophages	Suggests a possible association between appendectomy, immune remodeling, and CRC outcome

CRC, colorectal cancer.

## 4 Immune and microbiota mechanisms underlying the association

Whether appendectomy induces durable microbiota remodeling has become one of the most important mechanistic questions in this field. Cai and colleagues reported that appendectomy was associated with altered bacterial and fungal community structure, with particularly durable changes in the fungal compartment (Cai et al., 2021). Yap and colleagues later summarized the growing evidence linking post appendectomy microbiota changes to CRC related outcomes, emphasizing that the safe house concept is increasingly supported by both human and experimental evidence (Yap et al., 2024). At present, most human data support associations, whereas several downstream mechanistic links are inferred from animal, metagenomic, or translational studies rather than confirmed as direct causal pathways in patients. These observations are important because the broader CRC literature provides a biologically plausible framework linking dysbiosis to carcinogenesis. Reviews by Sears, Garrett, Brennan, Wong, Janney, Cheng, Tilg, and Montalban Arques have outlined multiple routes through which the gut microbiota can drive tumorigenesis, including inflammatory amplification, oncogenic metabolite production, epithelial barrier damage, genotoxicity, innate immune activation, and immune suppression (Sears and Garrett, 2014; Garrett, 2015; Brennan and Garrett, 2016; Sun and Kato, 2016; Raskov et al., 2017; Tilg et al., 2018; Montalban-Arques and Scharl, 2019; Wong and Yu, 2019; Cheng et al., 2020; Janney et al., 2020).

Among the most important microbial players is F. nucleatum. Kostic and colleagues showed that this bacterium promotes intestinal tumorigenesis and selectively expands intratumoral myeloid cells, thereby shaping a pro inflammatory tumor immune microenvironment (Kostic et al., 2013). Rubinstein and colleagues demonstrated that the same organism can activate epithelial oncogenic signaling through E cadherin and beta catenin pathways (Rubinstein et al., 2013). Human studies then extended this work by linking F. nucleatum burden to T cell patterns, patient survival, and chemoresistance (Mima et al., 2015; Mima et al., 2016; Yu et al., 2017). Together, these data establish that microbial composition can reshape tumor immunity, not merely coexist with it.

If the appendix contributes to the maintenance or redistribution of specific microbial communities, then appendectomy could alter which microbes gain access to the mucosa and which immune programs dominate in the tumor microenvironment. This is especially important for myeloid biology. Shi and colleagues provided important mechanistic evidence in experimental systems, together with supportive observational findings in humans. Their study suggested that appendectomy was associated with enrichment of CRC-promoting bacterial taxa and depletion of potentially beneficial commensals in human observations, while experimental models further supported links with perturbed microbial interaction networks, epithelial barrier dysfunction, and enhanced tumorigenesis. However, these findings should be interpreted cautiously, because the human data remain observational and the downstream biological links are not yet established as direct causal mechanisms in patients (Shi et al., 2023). A separate population based Asian cohort also reported altered later digestive cancer risk patterns after appendectomy, although such observational findings remain susceptible to bias and should not be over interpreted as causal proof (Hu et al., 2024).

The immune consequences of these ecological changes may be even more relevant than incidence itself. Liu and colleagues reported that prior appendectomy was associated with poorer CRC prognosis and with depletion of M1 like macrophages, suggesting a possible link between appendectomy and impaired antitumor immune activity (Liu et al., 2024). This is highly plausible in light of the broader literature on tumor associated macrophages in CRC. Reviews and original studies have consistently shown that macrophage biology in colorectal tumors is complex and context dependent. Total macrophage infiltration does not always predict poor outcome, whereas immunosuppressive phenotypes, especially M2 like programs and adverse spatial localization, are more consistently associated with invasion, metastasis, and resistance (Li et al., 2020; Inagaki et al., 2021; Wang et al., 2021; Li et al., 2022). Therefore, the effect of appendectomy may depend less on absolute macrophage number than on the balance of macrophage states and niches it favors.

**Figure 1:**
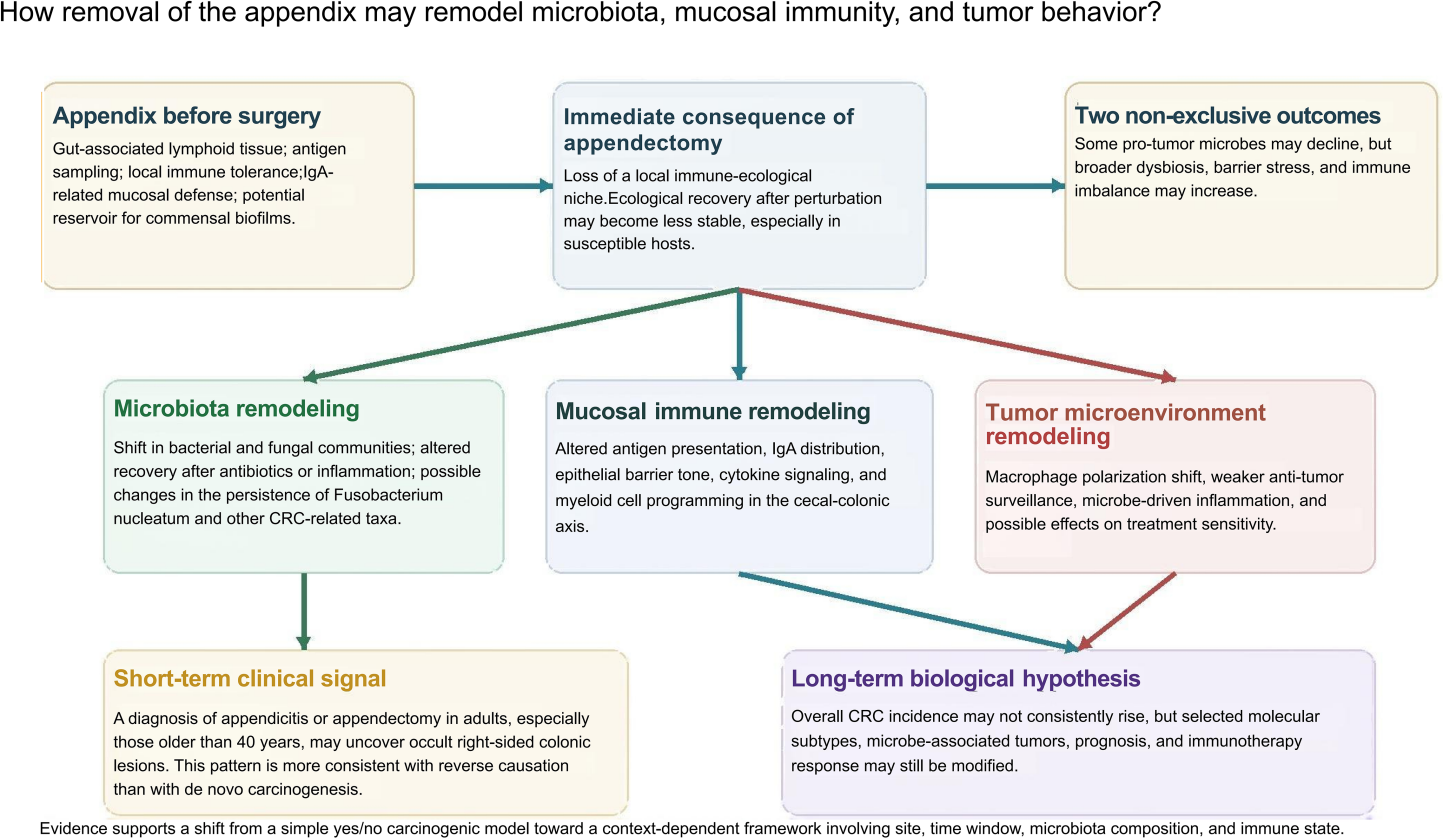
Proposed microbiota and immune mechanisms linking appendectomy to CRC. The appendix may function as a microbiota-associated and immune-active niche that contributes to local antigen sampling, IgA-related mucosal responses, microbial biofilm maintenance, and ecological resilience. Appendectomy may disrupt this balance by altering microbial communities, weakening epithelial barrier integrity, and reshaping local inflammatory and myeloid-cell programs. These changes may influence colorectal cancer biology in a context-dependent manner, affecting short-term diagnostic signals, long-term subtype-specific risk, tumor-associated macrophage states, and treatment response.

This framework may help explain why appendectomy does not appear to increase overall CRC incidence consistently, while it may still be associated with differences in the behavior of tumors that arise in selected settings. In this context, appendectomy should not be interpreted as a universal initiator of cancer, but rather as a potential modifier of host-microbiota interaction and tumor immune ecology. Inflammatory network biology provides a useful broader framework for this interpretation. Lasry and colleagues summarized how innate immune activation, inflammatory cytokine circuits, and myeloid programs can contribute to colorectal carcinogenesis (Lasry et al., 2016). If appendectomy contributes to disturbed mucosal immune tolerance, altered barrier function, and changes in microbial exposure, it could influence the biological context in which colorectal neoplasia is initiated, selected, or clinically expressed (**Figure 1**).

## 5 Discussion

The current evidence does not justify telling patients that appendectomy causes CRC. A more accurate interpretation is that appendectomy is not a confirmed determinant of increased long term overall CRC incidence, and that much of the excess risk observed shortly after appendicitis or surgery is more plausibly explained by reverse causation and detection bias. This is especially true in older adults and in right sided lesions, where appendicitis may reflect an already existing occult colonic abnormality rather than a future carcinogenic consequence of surgery. This interpretation should also be viewed in the context of current age-based CRC screening guidance, which recommends routine screening for average-risk adults beginning at age 45 years, while supporting individualized evaluation in selected higher-risk clinical settings (Issaka et al., 2023).

At the same time, the conclusion that appendectomy is irrelevant would also be too simplistic. The appendix has genuine immune and microbial functions, and its removal may not affect all tumors in the same way. Instead, the consequences are likely to be context dependent and may vary according to host background, anatomy, microbiota composition, and tumor subtype. The lower long term incidence of F. nucleatum positive CRC after appendectomy reported by Kawamura and colleagues is a striking example of this subtype specific perspective (Kawamura et al., 2025). Similarly, the reported association between prior appendectomy, altered macrophage-related immune states, and poorer prognosis in established CRC should currently be interpreted as an emerging translational observation rather than a confirmed clinical mechanism. These findings raise the possibility that appendectomy may influence tumor immunity in selected settings, but further validation is still needed (Cavnar et al., 2017; Yang et al., 2020; Liu et al., 2024).

From an immunology perspective, this topic is valuable precisely because it reframes a classic surgical history within the broader framework of host microbe interaction. Appendectomy should not merely be recorded as a past operation. It may represent a long term biological variable capable of influencing microbial resilience, mucosal tolerance, myeloid programming, and the immune microenvironment of CRC (Randal Bollinger et al., 2007; Cai et al., 2021; Shi et al., 2023; Liu et al., 2024; Yap et al., 2024). This possibility is especially relevant in microsatellite stable CRC, where response to immune checkpoint blockade remains limited and where both microbiota and myeloid cells are increasingly recognized as major determinants of treatment outcome.

Future studies should therefore move beyond crude overall risk estimates. Prospective designs with lag period analyses are needed to separate short term diagnostic bias from true long term biological effects. Outcomes should be refined to include right sided tumors, precursor lesions, and microbe associated molecular subtypes. Multiomics approaches should define how appendectomy related ecological shifts translate into myeloid and T cell remodeling in the colorectal mucosa. Finally, translational studies should test whether prior appendectomy influences prognosis, chemotherapy response, or immunotherapy sensitivity in established CRC. Only when epidemiology, microbiology, and immunology are integrated within a single framework will the true place of appendectomy in CRC biology become clear.
